# Placental Pathology of COVID-19 with and without Fetal and Neonatal Infection: Trophoblast Necrosis and Chronic Histiocytic Intervillositis as Risk Factors for Transplacental Transmission of SARS-CoV-2

**DOI:** 10.3390/v12111308

**Published:** 2020-11-15

**Authors:** David A. Schwartz, Denise Morotti

**Affiliations:** 1 Department of Pathology, Medical College of Georgia, Augusta University, Augusta, GA 30912, USA; 2 Pathology Unit and Medical Genetics Laboratory, ASST Papa Giovanni XXIII, 24127 Bergamo, Italy; dmorotti@asst-pg23.it

**Keywords:** COVID-19, maternal COVID-19 infection, fetal COVID-19 infection, placental COVID-19 infection, trophoblast necrosis, chronic histiocytic intervillositis, placental risk factors, neonatal COVID-19 infection, vertical transmission, placental pathology, SARS-CoV-2, maternal-fetal COVID-19, transplacental COVID-19 infection

## Abstract

The mechanism(s) by which neonates testing positive for coronavirus disease 2019 (COVID-19) acquire their infection has been largely unknown. Transmission of the etiological agent, SARS-CoV-2, from mother to infant has been suspected but has been difficult to confirm. This communication summarizes the spectrum of pathology findings from pregnant women with COVID-19 based upon the infection status of their infants and addresses the potential interpretation of these results in terms of the effects of SARS-CoV-2 on the placenta and the pathophysiology of maternal-fetal infection. Placentas from pregnant women with COVID-19 and uninfected neonates show significant variability in the spectrum of pathology findings. In contrast, placentas from infected maternal-neonatal dyads are characterized by the finding of mononuclear cell inflammation of the intervillous space, termed chronic histiocytic intervillositis, together with syncytiotrophoblast necrosis. These placentas show prominent positivity of syncytiotrophoblast by SARS-CoV-2, fulfilling the published criteria for transplacental viral transmission as confirmed in fetal cells through identification of viral antigens by immunohistochemistry or viral nucleic acid using RNA in situ hybridization. The co-occurrence of chronic histiocytic intervillositis and trophoblast necrosis appears to be a risk factor for placental infection with SARS-CoV-2 as well as for maternal-fetal viral transmission, and suggests a potential mechanism by which the coronavirus can breach the maternal-fetal interface.

## 1. Introduction

Shortly after the recognition of coronavirus disease 2019 (COVID-19) in Wuhan, China in December 2019, there were reports of infections occurring in pregnant women in this and neighboring regions [1,2,3,4,5]. Clinical disease in the large majority of these mothers was non-existent or mild [1,6], with only rare instances of significant maternal complications. Similar to such previous coronavirus diseases as severe acute respiratory syndrome (SARS), Middle East respiratory syndrome (MERS) [7], and other RNA respiratory viruses [8], there was no definitive mother-to-infant (vertical) transmission recognized at that time [8,9]. However, following the subsequent progression of the pandemic to involve countries in the Western Hemisphere, Europe, and the Middle East, the spectrum of reported clinical manifestations of the infection in pregnancy appeared to worsen, with pregnant women developing severe and critical pneumonia, thrombosis, cardiomyopathy, multiorgan disease, need for intensive care, and mechanical ventilation which, in a small number of cases, resulted in maternal deaths [10,11,12,13]. In addition to reports of increases in maternal morbidity and mortality, there were increasing descriptions of newborn infants who tested positive for SARS-CoV-2 (severe acute respiratory syndrome coronavirus 2), the causative agent of COVID-19 [14,15,16,17]. Analysis of pooled data from multiple studies indicated that only a small percentage of neonates delivered to pregnant women with COVID-19 tested positive for the virus [18]. However, it was difficult, if not impossible, to be certain of the source of these infections, whether they represented vertical infections, and if they did, how and when mother-to-infant transmission was occurring [19,20,21].

It is important to distinguish between intrauterine transplacental transmission of SARS-CoV-2 from an infected mother to her fetus from other mechanisms of vertical and neonatal infection. Schwartz et al. have proposed placental pathology criteria for the diagnosis of transplacental SARS-CoV-2 transmission between infected maternal-neonatal dyads [22]. These criteria are based upon the molecular identification of the virus on the fetal side of the placenta, such as in cells of the trophoblast or chorionic villous stroma, by demonstrating either viral antigens by immunohistochemistry or through detection of viral nucleic acid using RNA in situ hybridization or RNAscope methods. Based upon these criteria, there have been at least several neonates who appear to have acquired their COVID-19 infection in utero by transplacental transmission of the virus from an infected mother [23,24,25,26].

The pathology findings from placentas from pregnant women with COVID-19 have been variable—some reports describe no significant changes, some describe evidence of maternal of either fetal or maternal vascular malperfusion or both, and others describe inflammatory lesions including chronic histiocytic intervillositis, villitis, funisitis, and chorioamnionitis. Some authors have described a COVID-related placental spectrum of microscopic alterations, while others state that there are no specific findings of COVID-19 in placentas from infected women. The authors believe that, similar to other infectious diseases, the placental pathology can be, at least partially, the reflection of the passage of virus across the maternal-placental interface, and therefore cases of infected maternal-fetal dyads may have placental pathology findings that differ from those where there is no fetal or neonatal infection.

This communication provides an analysis of the spectrum of pathology findings from pregnant women with COVID-19 based upon the infection status of their infants and addresses the potential interpretation of these results in terms of the effects of SARS-CoV-2 on the placenta and the pathophysiology of maternal-fetal infection. It also examines the placental pathology findings in terms of placental and fetal infection with SARS-CoV-2 to determine if placental risk factors exist for developing intrauterine transplacental fetal infection.

## 2. Placental Pathology from Mothers with COVID-19 in the Absence of Neonatal Infection

The majority of placental pathology studies published to date have described placentas from pregnant women with COVID-19 who delivered neonates with no evidence of infection. In these non-transmitting cases, the authors believe that there are inherent limitations to the knowledge that can be gained, not only from the effects of the virus on the placental maternal interface but also on any placental factors that might be associated with transmission to the fetus.

During the epidemic of SARS-CoV-2 that occurred in Wuhan, China in early 2020, Chen and colleagues collected placentas from 3 pregnant women in the late 3rd trimester with mildly symptomatic COVID-19 at the Tongji Hospital [27]. In this first description of placental pathology from women infected with the novel coronavirus, the findings included varying degrees of increased perivillous fibrin and syncytial knotting, with no significant microscopic changes that could be causally related to maternal COVID-19 infection. Testing of placental tissues and the neonates using reverse transcriptase-polymerase chain reaction (RT-PCR) for SARS-CoV-2 were negative (Chen et al. 2020).

Shanes et al. [28] examined 16 placentas from pregnant women with COVID-19 at their hospital in Chicago, of whom 15 delivered live-born infants in the 3rd trimester, and one with an intrauterine fetal demise in the 2nd trimester. The neonates were tested for SARS-CoV-2 by nasopharyngeal and throat swabs using a SARS-CoV-2 RT-PCR assay, and all were found to be negative. The 15 3rd trimester placentas were compared with placentas from control populations unaffected by COVID-19. There were microscopic findings of maternal vascular malperfusion (MVM) in 12 placentas, higher than in the uninfected control groups. These MVM diagnoses included infarcts in the central (3/15) and peripheral (1/15) placental disc; villous agglutination (3/15); and accelerated villous maturation (2/15). Seven placentas (7/15) had decidual arteriopathy including 5 placentas with mural hypertrophy of membrane arterioles and 3 with atherosis and fibrinoid necrosis of maternal vessels. There were also findings of fetal vascular malperfusion (FVM) in 12 of the 15 placentas from mothers with COVID-19, but they were not increased in prevalence compared with the control groups. The occurrence of inflammatory lesions of the placentas from women with COVID-19 included acute inflammatory pathology (AIP) with histologic chorioamnionitis and umbilical arteritis in 1 placenta, and 2 placentas having chronic inflammatory pathology (CIP) consisting of low-grade chronic lymphocytic villitis and chronic deciduitis with plasma cells; however, these abnormalities were not increased compared with control placentas.

Baergen and Heller [29] enrolled 20 pregnant women in the 3rd trimester who tested positive for COVID-19 from their institution in New York City. At the time of presentation, 2 women were febrile and one had acute hypoxia and pneumonia. Another mother developed acute hypoxia and pneumonia 3 days after delivery. There were multiple maternal comorbidities in this cohort that included severe preeclampsia (2/20), Group B Streptococcus positive (3/20), and one case each with hypertension, postpartum hemorrhage, chronic diabetes, and hypothyroidism. None of the mothers required intensive care or ventilatory support. All of the newborn infants had Apgar scores of 8 or 9 at 1 min, and all infants had negative tests for COVID-19 using RT-PCR. Examination of the placentas revealed that the most frequent abnormality was low-grade fetal vascular malperfusion occurring in 45% (9/20) of cases. This consisted of intramural fibrin deposition present in 1 or 2 foci in 3 placentas, 2 placentas having only areas of villous stromal-vascular karyorrhexis, 3 placentas having multiple lesions, and a few placentas having nonocclusive intramural thrombi. Five (5/20) placentas showed maternal vascular malperfusion. There were 4 placentas having villitis of unknown etiology (VUE), 2 of which were high-grade, and one was associated with obliterative vasculopathy. One placenta demonstrated decidual vasculopathy.

In a retrospective cohort investigation of the placentas from 50 pregnant women diagnosed with COVID-19 at a hospital in New York and compared with an equal number of control placentas from uninfected women, Gulerson et al. [30] found that there were no statistically significant differences in the pathology findings between the two groups. All of the neonates from mothers with COVID-19 were negative for the virus by RT-PCR at 24 h of life.

Examining a cohort of pregnant women with COVID-19 in New York City, Prabhu et al. [31] evaluated 29 placentas from pregnant women with COVID-19 and 106 placentas from healthy, uninfected women. They found no statistically significant differences in the prevalence of maternal vascular malperfusion between the two groups. In contrast, abnormalities of fetal vascular malperfusion, consisting of thrombi in the fetal vessels, were more frequently present in the placentas of mothers with COVID-19 (14/29) than in mothers without the infection (12/106), representing a highly statistically significant difference (*p* < 0.001). None of the 29 neonates from pregnant women with COVID-19 tested positive for SARS-CoV-2.

From Basel, Menter and colleagues [32] described placentas at 40 to 41 weeks gestation from 5 pregnant women with COVID-19. Two mothers were asymptomatic; 3 had symptoms in the antenatal period including 1 just prior to delivery. SARS-CoV-2 testing of breast milk, umbilical cord blood, and amniotic fluid were all negative. The placenta from the mother with symptoms just prior to delivery showed a prominent lymphohistiocytic inflammatory infiltrate which included chronic lymphohistiocytic villitis and intervillositis as well as vasculitis of fetal vessels and focal thrombosis. Immunohistochemistry revealed that the inflammatory infiltrate was composed mostly of CD8-positive cytotoxic T-cells together with a mild increase of CD68-positive macrophages and scarce CD4-positive T-cells and plasma cells. There were also findings of maternal vascular malperfusion consisting of infarctions, increased intervillous fibrin, and intervillous thrombosis, as well as fetal vascular malperfusion consisting of thrombi in chorionic vessels with vasculitis. SARS-CoV-2 was identified in the decidua by in situ hybridization. The other 4 placentas showed variable findings of maternal vascular malperfusion; 1 of these 4 also had evidence of fetal vascular malperfusion, and another had prominent acute chorioamnionitis and focal chronic villitis. All 5 of the neonates tested negative for COVID-19 by RT-PCR and their clinical course was unremarkable.

Kuhrt et al. described a single case report of a 32-year-old pregnant woman with COVID-19 and a monochorionic diamniotic twin pregnancy who developed a placental abruption at 32^6^ weeks gestation requiring emergency cesarean section [33]. The placenta was reported to have accelerated maturation of villi and other findings of mild hypoperfusion. Both twin infants had negative testing by RT-PCR for SARS-CoV-2 on day of life (DOL) 3 and 5.

In a case report from the Netherlands by Mongula et al. [34], a 27-year-old pregnant woman was diagnosed with COVID-19 at 31^4^ weeks gestation following the onset of fever, headache, shortness of breath, malaise, coughing, and decreased fetal movement. She subsequently developed anemia, thrombocytopenia, and increases in lactate dehydrogenase (LD) and alanine aminotransferase (ALT). A vaginal swab was positive for SARS-CoV-2 by RT-PCR. An emergency cesarean section was performed and resulted in a newborn with APGAR scores of 3, 6, and 10 at 1, 5, and 10 min, respectively. The placenta showed increased fibrin deposition extending transmurally from the chorionic plate down to the decidua. The authors describe the intervillous space reduced by 30 to 50%, as well as trophoblast necrosis and extensive intervillositis composed of histiocytes and granulocytes. A swab taken from the fetal side of the placenta was positive for SARS-CoV-2 using RT-PCR. Immunohistochemistry revealed the presence of SARS-CoV-2 immunoreactive chorionic villous stromal cells and trophoblast. The neonate was negative for COVID-19.

Hsu and colleagues described a case report of a pregnant woman with COVID-19 who had mild symptoms of COVID-19 prior to her delivery at 40^4^ weeks gestation of a healthy infant who tested negative for the virus using RT-PCR [35]. The infant’s placenta showed chronic villitis and hypertrophic arteriopathy. Immunohistochemistry using an antibody to SARS-CoV-2 nucleocapsid antigen showed positive staining in chorionic villus endothelial cells and, rarely, in trophoblast.

At a single institution in New York City, Smithgall et al. compared placentas from 51 pregnant women having COVID-19 with 25 placentas from pregnant women who were uninfected [36]. There were no specific microscopic changes related to SARS-CoV-2 infection observed in the placentas of women with COVID-19. Although a variety of pathological findings were identified in placentas from women with COVID-19, there were only 2 abnormalities of maternal and fetal vascular malperfusion that were more likely to occur in the placentas of infected women compared with placentas from uninfected women—these were villous agglutination and subchorionic thrombi. Evaluation of placentas for SARS-CoV-2 using immunohistochemistry and in situ hybridization showed no evidence of placental infection. All of the newborn infants born to women with COVID-19 tested negative for the virus.

In New York City, Algarroba and colleagues reported a pregnant woman who was severely ill with COVID-19. She presented to the hospital at 28^4^ weeks of gestation and required intubation [37]. A cesarean section was performed to enhance maternal and fetal survival; PCR was not performed on the placenta or amniotic fluid. Both mother and infant survived, with the neonate testing negative for SARS-CoV-2 on DOL 2 and 3. The placenta demonstrated decidual vasculopathy and villous edema. Electron microscopy revealed rare, individual virions in cells of the syncytiotrophoblast as well as in fibroblastic-type cells in the placental villi [37]. In response to a published inquiry [38], additional evaluation of the placenta was performed which revealed that the virus was detected using immunogold electron microscopy; immunohistochemistry was positive for SARS-CoV-2 antigen; and the virus was identified in the placenta using RT-PCR primers [39]. However, the localization of the virus in the placenta using RT-PCR and immunohistochemistry was not described.

In 2 hospitals in Boston, Hecht et al. examined placentas from 19 pregnant women having COVID-19 in the peripartum period and compared the findings with 3 sets of control placentas from women who were uninfected with the coronavirus [40]. The authors found that there were no specific gross or microscopic findings in placentas from women with COVID-19. Only 1 of the neonates from mothers with COVID-19 tested positive for SARS-CoV-2 after delivery. Immunohistochemical evaluation revealed that 18 of 19 placentas were negative for viral antigen, and RNA in situ hybridization was negative in 17 placentas. The authors found rare, weak expression of viral RNA using in situ hybridization in the maternal endothelium of the decidua parietalis in several cases. In one placenta there was strong positive staining of the syncytiotrophoblast by immunohistochemistry and RNA in situ hybridization. In another placenta, there was weak staining for the virus in trophoblast cells only with in situ hybridization. There was no fetal vascular malperfusion present in the 2 placentas with evidence of the virus, although one had findings of mild maternal vascular malperfusion.

## 3. Placental Pathology with Evidence of Intrauterine Transplacental Maternal-Fetal COVID-19 Transmission

**Figure 1:**
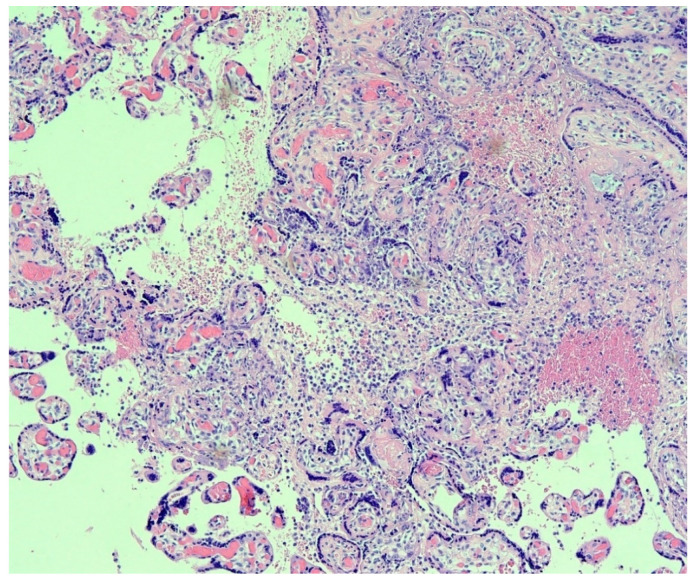
Placenta from a maternal-neonatal dyad with COVID-19. There is prominent chronic histiocytic intervillositis present in which the inflammatory cells in the intervillous space stained positive with the CD68 antibody. Necrosis of the syncytiotrophoblast layer on chorionic villi is visible even at this magnification. The findings, in this case, were indicative of intrauterine transplacental transmission of SARS-CoV-2. This placenta was described in [23]. Hematoxylin & eosin, ×10 magnification.

**Figure 2:**
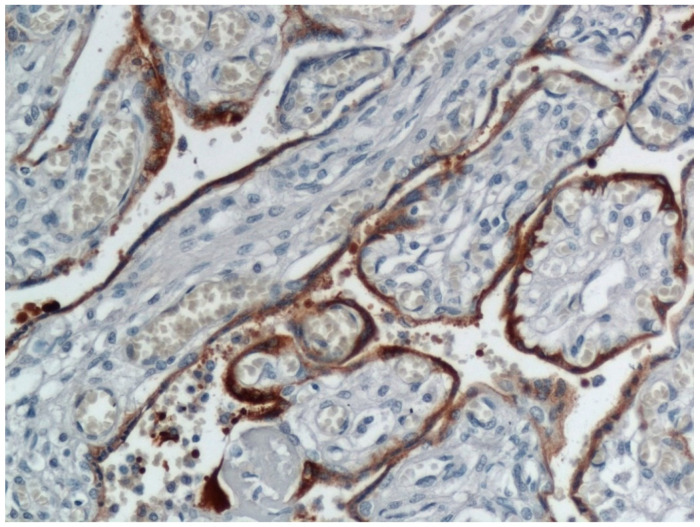
Placenta from the same maternal-neonatal dyad as in Figure 1 in which there was intrauterine maternal-fetal transmission of COVID-19. The confluent areas of brown staining at the periphery of the chorionic villi represents trophoblast infection identified by immunohistochemistry using an antibody to SARS-CoV-2 nucleocapsid protein. Positive staining is also present in inflammatory cells in the intervillous space. This placenta was described in [23]. ×20 magnification.

**Figure 3:**
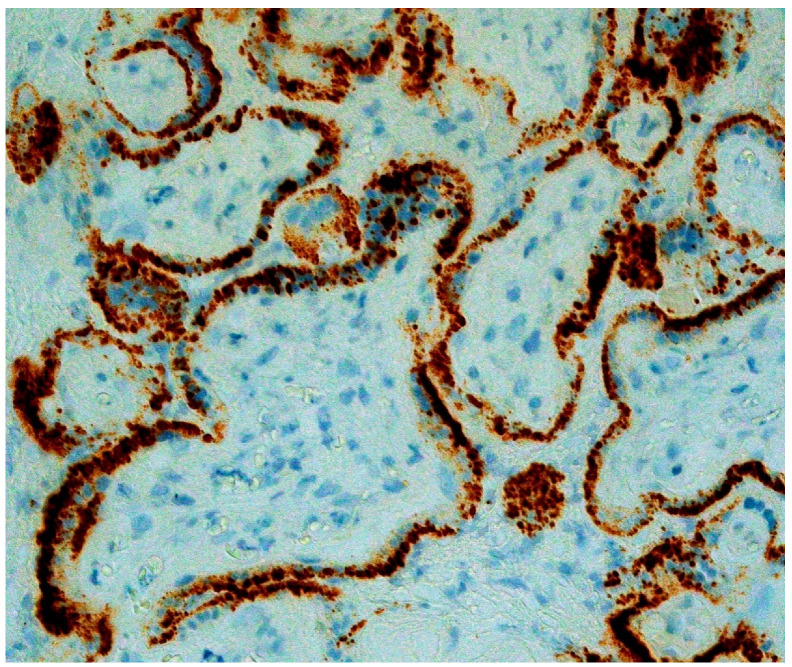
RNAscope for COVID-19 spike protein viral RNA (brown dots) staining positive within the syncytiotrophoblast of multiple chorionic villi. The trophoblast in these villi are intact and have not yet undergone necrosis. There is an intense viral load present. This placenta was described in [23]. ×40 magnification.

In a significant report from Italy, Patanè and colleagues evaluated 22 pregnant women with COVID-19 giving birth at the Papa Giovanni XXIII Hospital in Bergamo, Italy [23]. Two of the neonates tested positive for the novel coronavirus using RT-PCR from a nasal swab—one immediately after delivery, and the other testing negative at birth but who tested positive at DOL 7 after being isolated. Neither neonate had worrisome symptoms beyond some mild feeding difficulties after delivery. The placentas from both infants were abnormal, and both demonstrated chronic histiocytic intervillositis (Figure 1) in which the intervillous spaces contained numerous CD68-positive macrophages. In situ hybridization using RNAscope methods for the spike protein mRNA of SARS-CoV-2 as well as immunohistochemistry for SARS-CoV-2 antigens showed extensive positive staining in the syncytiotrophoblast of both placentas (Figure 2 and Figure 3) from the neonates testing positive for COVID-19. This represented the first report of pregnant women and their neonates testing positive for COVID-19 in which the fetal tissues of the placenta were found to be infected using molecular methods. These findings by Patanè et al. [23] were consistent with the published criteria of Schwartz et al. [22] for the identification of intrauterine transplacental maternal-fetal infection.

A case report from Toronto by Kirtsman et al. described a pregnant woman with familial neutropenia, recurrent bacterial infections, and gestational diabetes who developed symptomatic COVID-19 just prior to delivery and underwent cesarean section due to worsening coagulopathy close to 36 weeks gestation [26]. The infant had good Apgar scores −9 and 9 at 1 and 5 min, respectively. Nasopharyngeal swabs were taken on the day of birth and days 2 and 7 following delivery, and all were positive for SARS-CoV-2 using RT-PCR. Plasma from the infant was positive on DOL 4, and the neonatal stool was positive on DOL 7. Swabs of the placenta taken from both maternal and fetal sides, as well as a specimen of placental tissue, were all tested for SARS-CoV-2 and found to be positive. The placenta showed extensive and widespread areas of early infarction, as well as a prominent inflammatory, infiltrate that was almost exclusively confined to the intervillous space [41] (personal communication with W. Tony Parks MD). The syncytiotrophoblast demonstrated early but extensive necrosis.

Sisman et al. described a case report of a pregnant woman with Class B diabetes and obesity who developed COVID-19 at 34 weeks gestation [41]. She delivered a neonate with normal Apgar scores, but who developed indirect hyperbilirubinemia 24 h following birth, became febrile, and had respiratory distress on DOL 2 that required oxygen via a nasal cannula. Nasopharyngeal swabs performed 24 and 48 h after delivery were positive for SARS-CoV-2 by RT-PCR, which remained positive up to DOL 14. The infant was weaned to room air by DOL 5 and was discharged home on DOL 21. The infant’s placenta was large-for-gestational age. Microscopic findings included patchy chronic histiocytic intervillositis which was positive using the CD68 antibody, together with villous necrosis and karyorrhexis. Additional findings included focal basal chronic villitis, a focal parabasal infarct, and evidence of meconium in the fetal membranes. Immunohistochemistry for SARS-CoV-2 nucleocapsid protein was positive in the cytoplasm of the syncytiotrophoblastic cells. Electron microscopy revealed structures that consistent with viral particles clustered within membrane-bound cisternal spaces in syncytiotrophoblastic cells.

Vivanti and associates reported a pregnant primigravida admitted to a French hospital at 35^+2^ weeks gestation with fever, severe cough, and abundant expectorations [42]. She was found to be positive for SARS-CoV-2 in her blood and from swabs of the nasopharynx and vagina. Amniotic fluid taken at the time of the cesarean section also tested positive for SARS-CoV-2 by RT-PCR. The neonate required resuscitative measures, and both blood and non-bronchoscopic bronchoalveolar lavage fluid were collected for viral testing and found to be positive for the E and S genes of SARS-CoV-2. Both nasopharyngeal and rectal swabs were taken at 1 h after birth and on DOL 3 and 18—all were positive by RT-PCR for the two SARS-CoV-2 genes. Placental tissue was found to be positive for SARS-CoV-2 by RT-PCR at levels higher than in maternal and fetal blood and the amniotic fluid. Microscopic examination of the placenta revealed diffuse perivillous fibrin deposition and infarction, as well as acute and chronic intervillositis. The inflammatory infiltrate in the intervillous space consisted of histiocytes and neutrophils – the histiocytes stained positively with the anti-CD68 antibody. The syncytiotrophoblast stained intensely positive using immunohistochemistry and an antibody to SARS-CoV-2 N-protein. Our examination of the published photomicrographs showed trophoblast necrosis associated with the chronic histiocytic intervillositis.

**Figure 4:**
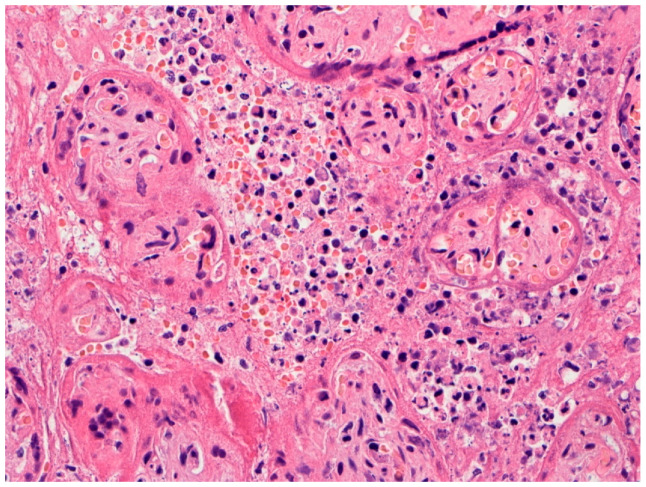
Higher magnification of placenta from a pregnant woman with COVID-19 in a case of transplacental SARS-CoV-2 fetal infection. Chronic histiocytic intervillositis is present in which the intervillous space is crowded with mononuclear inflammatory cells and necrotic cell debris and fibrin. Trophoblast necrosis can be seen on the surface of some chorionic villi. This placenta is described in [24]. Hematoxylin & eosin, ×20 magnification. Photograph courtesy of Fabio Facchetti, MD, Ph.D. of the University of Brescia, Italy.

In Italy, Fachetti et al. screened placentas from 15 pregnant women with COVID-19 using immunohistochemistry for SARS-CoV-2 nucleocapsid antigen to identify infected placentas [24]. One placenta tested positive for the virus and underwent analysis in greater detail. It was from a 29-year-old woman hospitalized at 37^5^ weeks gestation due to fever and worsening idiopathic thrombocytopenia. Vaginal delivery resulted in a healthy-appearing boy, but 24 h later he developed fever, breathing difficulty, vomiting, abdominal distension, hypotonia, and cutaneous mild erythema. He had radiological evidence of interstitial pneumonia and required intubation and mechanical ventilation. Just after birth his nasopharyngeal swab for SARS-CoV-2 by RT-PCR was inconclusive, but repeat testing at 36 and 72 h, and ultimately 17 days, following delivery was positive for the coronavirus. His condition improved and he was discharged on DOL 18. The placenta showed histiocytic-neutrophilic intervillositis (Figure 4). The chorionic villi that were involved by inflammation demonstrated thinning, discontinuity, and necrosis of the syncytiotrophoblast cell layer that was confirmed by immunohistochemistry for cytokeratin. There was evidence of fetal vascular malperfusion consisting of avascular and fibrotic villi and stroma-vascular karyorrhexis, and prominent perivillous fibrin deposition. Accelerated villous maturation and chorangiosis were also present. Immunohistochemistry revealed increased Hofbauer cells which expressed programmed death-ligand 1 (PD-L1). Scattered neutrophil extracellular traps (NETs) were identified by immunofluorescence. RNA in situ hybridization for SARS-CoV-2 demonstrated strong expression of viral RNA in the syncytiotrophoblast, as well as in scattered CD14^+^ intervillous mononuclear cells. Immunohistochemistry for SARS-CoV-2 spike and nucleocapsid proteins showed the syncytiotrophoblast to be positive for both proteins. Interestingly, the authors observed that anti-nucleocapsid staining was homogenously strong diffusely in the placental parenchyma, while immunoreactivity to the S-protein was variable but particularly strong in areas with abundant intervillous inflammation. Ultrastructural analysis demonstrated coronavirus-like particles present in chorionic villi within the cytoplasm of syncytiotrophoblast cells and also in the endothelium of fetal capillaries and fibroblasts. Particles consistent with the appearance of coronaviruses were seen within the cytoplasm of cells within the capillary lumina, which were likely monocytes.

## 4. Placental Pathology with Intrauterine Fetal Demise from Mothers with COVID-19

Baud et al. described the delivery of a stillborn fetus from a French woman with a mild form of COVID-19 at approximately 19 weeks gestation [43]. The amniotic fluid and vaginal swabs taken during labor, and swabs taken from the axillae, mouth, meconium, and blood from the fetus obtained within minutes of birth, all tested negative for SARS-CoV-2 using RT-PCR. An autopsy was performed, and biopsies of fetal lung, liver, and thymus biopsies were also negative for the coronavirus. The fetal surface of the placenta was disinfected, and the swabs and tissue biopsies that were taken tested positive for SARS-CoV-2, although the localization of the virus could not be established using this methodology. Microscopic examination of the placenta showed evidence of mild maternal vascular malperfusion (increased syncytial knots villous fibrin) and an ascending bacterial infection including acute subchorionitis and acute funisitis.

Hosier et al. [44] described a 33-year-old G3P1 pregnant woman presenting to the hospital with a 10-day history of symptoms of COVID-19 at 22 weeks gestation. Her nasopharyngeal swab was positive for SARS-CoV-2 by RT-PCR at the time of admission. She was found to have hypertension, low platelets, proteinuria, and elevated transaminases, leading to a diagnosis of severe preeclampsia. The patient also had severe deficiencies in clot formation and underwent treatment with multiple blood products for coagulopathy. To reduce potential morbidity or mortality, she decided to terminate this pre-viable pregnancy. Following the delivery of the fetus, quantitative PCR for SARS-CoV-2 revealed that the placenta and umbilical cord were positive for the coronavirus and that fetal lung and cardiac tissues were negative for infection. The placenta demonstrated chronic histiocytic intervillositis in which the inflammatory infiltrate was composed of macrophages and T lymphocytes which were confirmed by immunohistochemistry for CD68 and CD3. SARS-CoV-2 was present predominantly in the syncytiotrophoblast using both immunohistochemistry with antibody to the spike protein and RNA in situ hybridization. Electron microscopy revealed coronavirus particles within the cytoplasm of chorionic villus cells including syncytiotrophoblast, cytotrophoblast, fibroblasts, and capillary endothelial cells.

In Brazil, Richtman et al. reported the occurrence of 5 pregnant women with symptomatic COVID-19 who had fetal deaths occurring on days 1 through 22 of their illness [45]. These intrauterine fetal demises occurred from 21 to 38 weeks gestation. All 5 placentas demonstrated the presence of acute chorioamnionitis, with 2 placentas also having acute villitis and intervillositis and increased fibrin deposition. Samples taken from the placentas of 2 cases were positive for SARS-CoV-2 by RT-PCR. The fetuses were not tested for COVID-19.

Pulinx and colleagues reported a 30-year-old pregnant woman in Belgium with dichorionic diamniotic twins at 22 weeks gestation who was diagnosed with mild symptomatic COVID-19 at the Emergency Department and discharged [46]. She returned 2 weeks later complaining of back and low abdominal pain, and no symptoms referable to her COVID-19 infection. She gave birth to two stillborn preterm fetuses. Maternal blood, amniotic fluid, and placental tissue samples all tested positive for SARS-CoV-2 using RT-PCR; samples of both amniotic sacs tested negative for the coronavirus. Microscopic examination of the twin placentas demonstrated chronic intervillositis composed of aggregated histiocytes and cytotoxic T lymphocytes in the intervillous space which were confirmed by immunohistochemistry for CD68, CD3, and CD8. There was also extensive intervillous fibrin deposition with necrosis of the surrounding villi. Positive staining of the syncytiotrophoblast cells was present as confirmed by immunohistochemistry using the SARS-CoV-2 antibody.

## 5. Trophoblast Necrosis Together with Chronic Histiocytic Intervillositis Appears to Be a Risk Factor for Placental Infection and Maternal-Fetal Transmission of COVID-19

The maternal-fetal interface is a highly complex structure that is necessary to maintain balance and control between biochemical, hormonal, and maternal immunological factors that are necessary for fetal development and a normal pregnancy to proceed. The placenta is formed of fetal-derived cells and is the primary barrier between the mother and fetus during pregnancy. It is unique among eutherian organisms in permitting intimate contact between the fetal-derived trophoblastic cells and maternal cells. The maternal-fetal interface consists of trophoblast progenitor cells which ultimately differentiate into cytotrophoblasts, having a proliferative capability, and the syncytiotrophoblast, which are multinucleated and fused terminally differentiated cells. Syncytiotrophoblasts line the surface of the chorionic villous trees and are in direct contact with circulating maternal blood in the intervillous space. The syncytiotrophoblast layer is strongly resistant to infection from numerous infectious agents, providing a critically important function in protecting the fetus against viruses and other pathogens. The mechanisms by which trophoblast provides these protective functions are not well understood but include physical properties and potential paracrine pathways and formation of antimicrobial peptides such as toll-like receptors, defensin, and nucleotide-binding oligomerization domain (NOD) proteins. Enhancing its role as the key cellular layer of the intrinsic defense system of the placenta, syncytiotrophoblast is periodically regenerated, does not contain periodic gap junctions, and has a uniquely dense cytoskeletal network that provides a protective brush border at the apical surface. In addition, cytotrophoblast cells can also play a defensive role, and the basement membrane zone lying beneath these cells also form an additional physical barrier [47,48,49,50].

The mechanism(s) by which viral pathogens can penetrate the protective syncytiotrophoblast cell layer to reach the underlying villous core are not well-known. However, one potential mechanism would be to disrupt the normally continuous syncytiotrophoblast layer through necrosis. In the situation of a pathogen breaching the trophoblast layer, and then gaining access to the chorionic villous core containing Hofbauer cells, fibroblasts, fetal endothelial cells, and blood vessels, infection of the fetus could occur with potentially devastating consequences. The consistent observation discussed in this communication that trophoblast necrosis has occurred in placentas from pregnant women with COVID-19 who transmit the infection to their fetus, and that all of these placentas had evidence for SARS-CoV-2 infection of trophoblast confirmed by molecular pathology, is a potentially important finding in understanding how the virus can penetrate the maternal-fetal interface. The presence of a rare inflammatory placental lesion, chronic histiocytic intervillositis, is also associated with placental infection with SARS-CoV-2 and villous necrosis in these cases and is of potential significance in providing a mechanism for transplacental transmission of the virus and fetal infection. Although the number of placentas available for analysis that fulfill the criteria for intrauterine fetal infection is currently limited, the simultaneous co-occurrence of these two pathology findings appears to be risk factors for placental infection with SARS-CoV-2, as well as for subsequent intrauterine maternal-fetal viral transmission. A communication in greater detail that analyzes these pathology findings and their relationship with placental and fetal infection is currently in preparation.

## 6. Discussion

There have been rapid and significant advances made in understanding the clinical spectrum of SARS-CoV-2 infection in pregnant women and their infants since the beginning of the COVID-19 pandemic, but there still remains much to learn. In addition, increasing numbers of global cohort studies of pregnant women with COVID-19 that examine neonatal outcomes have demonstrated that a small percentage of newborn infants will test positive for SARS-CoV-2 following birth. In a recent meta-analysis of 74 publications, there were a total of 176 neonates from around the world who tested positive for SARS-Cov-2 [51]. Of these, 174 were diagnosed using RT-PCR methods, and 2 diagnosed by the presence of IgM in newborn blood. Among these publications, the largest neonatal cohorts reported to test positive for SARS-CoV-2 were 19 newborns in Iran reported by Schwartz et al. [15], 15 newborns in Italy by Garazzino et al. [52], and 12 neonates in Great Britain described by Knight et al. [53]. An important metric that remains uncertain is the probability of a pregnant woman with COVID-19 having a neonate testing positive for the virus. Recent estimates of the prevalence of COVID-19 test positivity among these neonates vary, with studies reporting between less than 1% [54], 2 to 2.5% [55,56], 4% [57] and up to 5% [58] of newborns testing positive for the coronavirus.

As has occurred in previous epidemics of emergent infectious diseases such as with Ebola and Zika viruses [59,60], there has been a great concern for the potential of transmission of SARS-CoV-2 from the pregnant mother to her fetus via the transplacental route. Until recently, it was difficult to distinguish between the possible mechanisms that would account for neonates testing positive for COVID-19. These mechanisms include vertical infection from an infected mother such as ascending, transplacental, intrapartum, and postpartum infections, and neonatal infection arising from such other sources as transmission from healthcare personnel, family members, and environmental exposures. Proposed criteria by Schwartz et al. for identifying transplacental transmission of SARS-CoV-2 versus other mechanisms of infection using molecular analysis techniques of placental analysis will hopefully help to clarify this issue in many situations [22].

Cases of transplacental infection of fetuses with SARS-CoV-2 have now been described that meet the proposed criteria for this mechanism of transmission. These criteria include the use of immunohistochemistry and RNA in situ hybridization/RNAscope to identify the SARS-CoV-2 virus in cells on the fetal side of the placenta from maternal/neonatal dyads that test positive for COVID-19. In addition, some authors have also used ultrastructural analysis to identify the virus in fetal-derived placental tissues [24,41,44]. Fortunately, transplacental vertical infection appears to be uncommon. In a recent analysis, Raschetti and colleagues have estimated that among neonates testing positive for COVID-19, 70% are due to postpartum environmental exposure, and 30% result from prenatal vertical infection [51]. The authors expounded on these metrics by estimating that among all neonates testing positive for COVID-19, approximately 9% represented confirmed prenatal vertical infections −3.3% for intrapartum acquisition and 5.7% for congenitally (intrauterine) transmitted infections.

Placental analysis among neonates testing positive for COVID-19 has been revealing. This is in sharp contrast to the findings among transmitting placentas from the previous newly emergent viral disease, Zika virus, which revealed a paucity of pathology findings except for hyperplasia of chorionic villous stromal macrophages (Hofbauer cells) [61,62]. Although there are still limited numbers of placentas from pregnant women with COVID-19 that have been examined, several interesting trends in pathology findings can be noted between placentas that do not transmit the coronavirus and those that do transmit SARS-CoV-2 from mother to the fetus. There appears to be much greater variation in the spectrum of pathology findings among non-transmitting placentas than among those placentas associated with intrauterine SARS-CoV-2 transmission. Authors have reported a wide range of pathology features in placentas from women with COVID-19 whose infants test negative for COVID-19. Individual and combinations of abnormalities of maternal vascular malperfusion, fetal vascular malperfusion, inflammatory lesions including chorioamnionitis and villitis, and even absence of pathology findings have been reported in placentas from neonates testing negative for COVID-19. The significant variance in pathology findings among non-transmitting placentas has led to one article entitled “SARS-CoV-2 can infect the placenta and is not associated with specific placental histopathology” [40].

In contrast, there is greater uniformity of pathology features among the placentas from infected maternal-neonatal dyads. In addition to the occurrence of trophoblast necrosis, they all contain a descriptive reference to the same lesion—a predominantly histiocytic inflammatory cell infiltrate occupying the intervillous space, which is currently termed chronic histiocytic intervillositis. Given the variability that occurs when placentas are described by different pathologists having differing experiences, the concurrence in the diagnostic descriptions of the intervillous mononuclear inflammatory pathology in these transmitting placentas is significant.

Chronic histiocytic intervillositis (CHIV) is a placental lesion characterized by a diffuse infiltration of the intervillous space composed predominantly of mononuclear inflammatory cells termed histiocytes. It is a rare lesion, present in less than 1% of pregnancies. It was first identified in 1987 by Labarre and Mullen, who termed it massive chronic intervillositis, and described it as an intervillous infiltration of mononuclear cells in the placenta, with fibrin deposits and trophoblast necrosis [63]. They hypothesized that it might represent an extreme variant of villitis of unknown etiology. Since then, this lesion has been described by a variety of terms that include “massive chronic intervillositis”, “chronic histiocytic intervillositis”, “chronic intervillositis of unknown etiology”, “intervillitis”, “chronic intervillositis”, “chronic histiocytic intervillositis of unknown etiology”, “massive perivillous histiocytosis”, and “massive histiocytic chronic intervillositis [64,65]. CHIV can be accompanied by increased fibrin deposition [66], which in some cases is so extreme as to be classified as massive perivillous fibrin deposition [67,68]. The most important microscopic differential diagnosis for CHIV is the chronic stage of placental malaria, in which accumulations of histiocytes in the intervillous space also occur [69]. Although malaria co-exists in much of the world affected by COVID-19 [70], the pathology differential diagnosis is not difficult as placentas affected by malaria will also typically demonstrate *Plasmodium*-parasitized red blood cells and hemozoin pigment in the intervillous space, trophoblast necrosis is absent, and fibrin deposition is not prominent. Some cases of CHIV described prior to the COVID-19 pandemic occurred together with chronic villitis, which can result from infection with such TORCH infections as cytomegalovirus (CMV), toxoplasmosis, rubella, and syphilis [65]. However, in the cases of intrauterine transplacental transmission of SARS-CoV-2 described in this communication, there was no pathological component of villitis present and no evidence of CMV or other infectious agent besides SARS-CoV-2.

In addition to chronic histiocytic intervillositis and trophoblast necrosis, the transmitting placentas described in this report also demonstrate variable degrees of increased fibrin, including several with large areas of fibrin that are consistent with massive perivillous fibrin deposition. All of these cases demonstrated the presence of SARS-CoV-2 on the fetal side of the placenta, predominantly in the syncytiotrophoblast, but in one case with virus-positive cells in the intervillous space. Among some of the cases of premature stillbirths with placentas having fetal tissues infected with SARS-CoV-2, there were similar findings of mononuclear and histiocytic intervillositis, increased fibrin, and necrosis [44,45,46]. It is interesting that in one case there was chronic histiocytic intervillositis and trophoblast necrosis present together with markedly increased fibrin deposition and SARS-CoV-2 identified in the trophoblast and villous stroma [34], but in which the neonate tested negative for COVID-19. This may indicate that infection of the chorionic villi by SARS-CoV-2 does not immediately give rise to fetal infection, and that following a breach of the maternal-fetal interface in the placenta and trophoblast infection that a period of time is required for the virus to travel from the infected placenta and reach the fetus.

The co-occurrence of chronic histiocytic intervillositis and trophoblast necrosis in all of the placentas having syncytiotrophoblast infected with SARS-CoV-2, most of which are associated with transplacental fetal infection, leads to more questions than answers. No previous TORCH viral infection has been shown to be closely and repetitively associated with the chronic histiocytic intervillositis—why SARS-CoV-2? What additional risk factors exist for those small numbers of cases of transplacental transmission, and are they maternal, fetal, or placental in nature? Is the reason that so few pregnant women with COVID-19 have neonates with the infection based upon placental, viral, or maternal factors? What is the etiological association between chronic histiocytic intervillositis, trophoblast necrosis, and trophoblast infection with SARS-CoV-2? Are there specific host microbiological factors such as viral load or immunological factors such as the presence of maternal cytokine storm that are related to the placental pathology findings and transplacental transmission? As additional placentas become available for examination, future analyses of the pathology using routine and molecular methodologies will help clarify the nature of the maternal and fetal response to COVID-19 occurring during pregnancy, and how and under what circumstances the fetus may develop an infection.

## References

[1] Schwartz D.A. (2020). An analysis of 38 pregnant women with COVID-19, their newborn infants, and maternal-fetal transmission of SARS-CoV-2: Maternal coronavirus infections and pregnancy outcomes. Arch. Pathol. Lab. Med..

[2] Yu N., Li W., Kang Q., Xiong Z., Wang S., Lin X., Liu Y., Xiao J., Liu H., Deng D. (2020). Clinical features and obstetric and neonatal outcomes of pregnant patients with COVID-19 in Wuhan, China: A retrospective, single-centre, descriptive study. Lancet Infect. Dis..

[3] Zhu H., Wang L., Fang C., Peng S., Zhang L., Chang G., Xia S., Zhou W. (2020). Clinical analysis of 10 neonates born to mothers with 2019-nCoV pneumonia. Transl Pediatr..

[4] Chen H., Guo J., Wang C., Luo F., Yu X., Zhang W., Li J., Zhao D., Xu D., Gong Q. (2020). Clinical characteristics and intrauterine vertical transmission potential of COVID-19 infection in nine pregnant women: A retrospective review of medical records. Lancet.

[5] Zhang L., Jiang Y., Wei M., Cheng B.H., Zhou X.C., Li J., Tian J.H., Dong L., Hu R.H. (2020). Analysis of the pregnancy outcomes in pregnant women with COVID-19 in Hubei Province. Zhonghua Fu Chan Ke Za Zhi.

[6] Schwartz D.A. (2020). The effects of pregnancy on women with COVID-19: Maternal and infant outcomes. Clin. Infect. Dis..

[7] Schwartz D.A., Graham A.L. (2020). Potential maternal and infant outcomes from coronavirus 2019-nCoV (SARS-CoV-2) infecting pregnant women: Lessons from SARS, MERS, and other human coronavirus infections. Viruses.

[8] Schwartz D.A., Dhaliwal A. (2020). Infections in pregnancy with COVID-19 and other respiratory RNA virus diseases are rarely, if ever, transmitted to the fetus: Experiences with coronaviruses, HPIV, hMPV RSV, and influenza. Arch. Pathol. Lab. Med..

[9] Rasmussen S.A., Smulian J.C., Lednicky J.A., Wen T.S., Jamieson D.J. (2020). Coronavirus disease 2019 (COVID-19) and pregnancy: What obstetricians need to know. Am. J. Obstet. Gynecol..

[10] Galang R.R., Chang K., Strid P., Snead M.C., Woodworth K.R., House L.D., Perez M., Barfield W.D., Meaney-Delman D., Jamieson D.J. (2020). Severe coronavirus infections in pregnancy: A systematic review. Obstet. Gynecol..

[11] Takemoto M., Menezes M.O., Andreucci C.B., Knobel R., Sousa L., Katz L., Fonseca E.B., Magalhães C.G., Oliveira W.K., Rezende-Filho J. (2020). Maternal mortality and COVID-19. J. Matern. Fetal Neonatal Med..

[12] Juusela A., Nazir M., Gimovsky M. (2020). Two cases of coronavirus 2019-related cardiomyopathy in pregnancy. Am. J. Obstet. Gynecol. MFM.

[13] Ellington S., Strid P., Tong V.T., Woodworth K., Galang R.R., Zambrano L.D., Nahabedian J., Anderson K., Gilboa S.M. (2020). Characteristics of women of reproductive age with laboratory-confirmed SARS-CoV-2 infection by pregnancy status-United States, January 22–June 7, 2020. MMWR Morb. Mortal. Wkly. Rep..

[14] Zimmermann P., Curtis N. (2020). COVID-19 in children, pregnancy and neonates: A review of epidemiologic and clinical features. Pediatr. Infect. Dis. J..

[15] Schwartz D.A., Mohagheghi P., Beigi B., Zafaranloo N., Moshfegh F., Yazdani A. (2020). Spectrum of neonatal COVID-19 in Iran: 19 infants with SARS-CoV-2 perinatal infections with varying test results, clinical findings and outcomes. J. Matern. Fetal Neonatal Med..

[16] Dhir S.K., Kumar J., Meena J., Kumar P. (2020). Clinical features and outcome of SARS-CoV-2 infection in neonates: A systematic review. J. Trop. Pediatr..

[17] Sola A., Rodríguez S., Cardetti M., Dávila C. (2020). COVID-19 perinatal en América Latina [Perinatal COVID-19 in Latin America]. COVID-19 perinatal en América Latina [Perinatal COVID-19 in Latin America]. Rev. Panam. Salud Publica.

[18] Kotlyar A.M., Grechukhina O., Chen A., Popkhadze S., Grimshaw A., Tal O., Taylor H.S., Tal R. (2020). Vertical transmission of coronavirus disease 2019: A systematic review and meta-analysis. Am. J. Obstet. Gynecol..

[19] Yang Z., Liu Y. (2020). Vertical transmission of severe acute respiratory syndrome coronavirus 2: A systematic review. Am. J. Perinatol..

[20] Wang C., Zhou Y.H., Yang H.X., Poon L.C. (2020). Intrauterine vertical transmission of SARS-CoV-2: What we know so far. Ultrasound Obstet. Gynecol..

[21] Kimberlin D.W., Stagno S. (2020). Can SARS-CoV-2 infection be acquired in utero? More definitive evidence is needed. JAMA.

[22] Schwartz D.A., Morotti D., Beigi B., Moshfegh F., Zafaranloo N., Patane L. (2020). Confirming vertical fetal infection with COVID-19: Neonatal and pathology criteria for early onset and transplacental transmission of SARS-CoV-2 from infected pregnant mothers. Arch. Pathol, Lab. Med.

[23] Patanè L., Morotti D., Giunta M.R., Sigismondi C., Piccoli M.G., Frigerio L., Mangili G., Arosio M., Cornolti G. (2020). Vertical transmission of coronavirus disease 2019: Severe acute respiratory syndrome coronavirus 2 RNA on the fetal side of the placenta in pregnancies with coronavirus disease 2019-positive mothers and neonates at birth. Am. J. Obstet. Gynecol. MFM.

[24] Facchetti F., Bugatti M., Drera E., Tripodo C., Sartori E., Cancila V. (2020). SARS-CoV2 vertical transmission with adverse effects on the newborn revealed through integrated immunohistochemical, electron microscopy and molecular analyses of placenta. EBioMedicine.

[25] Schwartz D.A., Thomas K.M. (2020). Characterizing COVID-19 maternal-fetal transmission and placental infection using comprehensive molecular pathology. EBioMedicine.

[26] Kirtsman M., Diambomba Y., Poutanen S.M., Malinowski A.K., Vlachodimitropoulou E., Parks W.T., Erdman L., Morris S.K., Shah P.S. (2020). Probable congenital SARS-CoV-2 infection in a neonate born to a woman with active SARS-CoV-2 infection. CMAJ.

[27] Chen S., Huang B., Luo D.J., Li X., Yang F., Zhao Y., Nie X., Huang B.X. (2020). Pregnancy with new coronavirus infection: Clinical characteristics and placental pathological analysis of three cases. Zhonghua Bing Li Xue Za Zhi.

[28] Shanes E.D., Mithal L.B., Otero S., Azad H.A., Miller E.S., Goldstein J.A. (2020). Placental pathology in COVID-19. Am. J. Clin. Pathol..

[29] Baergen R.N., Heller D.S. (2020). Placental pathology in COVID-19 positive mothers: Preliminary findings. Pediatr. Dev. Pathol..

[30] Gulersen M., Prasannan L., Tam H.T., Metz C.N., Rochelson B., Meirowitz N., Shan W., Edelman M., Millington K.A. (2020). Histopathological evaluation of placentas after diagnosis of maternal SARS-CoV-2 infection. Am. J. Obstet. Gynecol. MFM.

[31] Prabhu M., Cagino K., Matthews K.C., Friedlander R.L., Glynn S.M., Kubiak J.M., Yang Y.J., Zhao Z., Baergen R.N., DiPace J.I. (2020). Pregnancy and postpartum outcomes in a universally tested population for SARS-CoV-2 in New York City: A prospective cohort study. BJOG.

[32] Menter T., Mertz K.D., Jiang S., Chen H., Monod C., Tzankov A., Waldvogel S., Schulzke S.M., Hösli I., Bruder E. (2020). Placental pathology findings during and after SARS-CoV-2 infection: Features of villitis and malperfusion. Pathobiology.

[33] Kuhrt K., McMicking J., Nanda S., Nelson-Piercy C., Shennan A. (2020). Placental abruption in a twin pregnancy at 32 weeks’ gestation complicated by coronavirus disease 2019 without vertical transmission to the babies. Am. J. Obstet. Gynecol. MFM.

[34] Mongula J.E., Frenken M., van Lijnschoten G., Arents N., de Wit-Zuurendonk L.D., Schimmel-de Kok A., van Runnard Heimel P.J., Porath M.M., Goossens S. (2020). COVID-19 during pregnancy: Non-reassuring fetal heart rate, placental pathology and coagulopathy. Ultrasound Obstet. Gynecol..

[35] Hsu A.L., Guan M., Johannesen E., Stephens A.J., Khaleel N., Kagan N., Tuhlei B.C., Wan X.F. (2020). Placental SARS-CoV-2 in a pregnant woman with mild COVID-19 disease. J. Med. Virol..

[36] Smithgall M.C., Liu-Jarin X., Hamele-Bena D., Cimic A., Mourad M., Debelenko L., Chen X. (2020). Third-trimester placentas of severe acute respiratory syndrome coronavirus 2 (SARS-CoV-2)-positive women: Histomorphology, including viral immunohistochemistry and in-situ hybridization. Histopathology.

[37] Algarroba G.N., Rekawek P., Vahanian S.A., Khullar P., Palaia T., Peltier M.R., Chavez M.R., Vintzileos A.M. (2020). Visualization of severe acute respiratory syndrome coronavirus 2 invading the human placenta using electron microscopy. Am. J. Obstet. Gynecol..

[38] Kniss D.A. (2020). Alternative interpretation to the findings reported in visualization of severe acute respiratory syndrome coronavirus 2 invading the human placenta using electron microscopy. Am. J. Obstet. Gynecol..

[39] Algarroba G.N., Hanna N.N., Rekawek P., Vahanian S.A., Khullar P., Palaia T., Peltier M.R., Chavez M.R., Vintzileos A.M. (2020). Confirmatory evidence of the visualization of severe acute respiratory syndrome coronavirus 2 invading the human placenta using electron microscopy. Am. J. Obstet. Gynecol..

[40] Hecht J.L., Quade B., Deshpande V., Mino-Kenudson M., Ting D.T., Desai N., Dygulska B., Heyman T., Salafia C., Shen D. (2020). SARS-CoV-2 can infect the placenta and is not associated with specific placental histopathology: A series of 19 placentas from COVID-19-positive mothers. Mod. Pathol..

[41] Sisman J., Jaleel M.A., Moreno W., Rajaram V., Collins R., Savani R.C., Rakheja D., Evans A.S. (2020). Intrauterine transmission of SARS-COV-2 infection in a preterm infant. Pediatr. Infect. Dis. J..

[42] Vivanti A.J., Vauloup-Fellous C., Prevot S., Zupan V., Suffee C., Do Cao J., Benachi A., De Luca D. (2020). Transplacental transmission of SARS-CoV-2 infection. Nat. Commun..

[43] Baud D., Greub G., Favre G., Gengler C., Jaton K., Dubruc E., Pomar L. (2020). Second-trimester miscarriage in a pregnant woman with SARS-CoV-2 infection. JAMA..

[44] Hosier H., Farhadian S.F., Morotti R.A., Deshmukh U., Lu-Culligan A., Campbell K.H., Yasumoto Y., Vogels C.B., Casanovas-Massana A., Vijayakumar P. (2020). SARS-CoV-2 infection of the placenta. J. Clin. Investig..

[45] Richtmann R., Torloni M.R., Oyamada Otani A.R., Levi J.E., Crema Tobara M., de Almeida Silva C., Dias L., Miglioli-Galvão L., Martins Silva P., Kondo M.M. (2020). Fetal deaths in pregnancies with SARS-CoV-2 infection in Brazil: A case series. Case Rep. Womens Health.

[46] Pulinx B., Kieffer D., Michiels I., Petermans S., Strybol D., Delvaux S., Baldewijns M., Raymaekers M., Cartuyvels R., Maurissen W. (2020). Vertical transmission of SARS-CoV-2 infection and preterm birth. Eur. J. Clin. Microbiol. Infect. Dis..

[47] Arora N., Sadovsky Y., Dermody T.S., Coyne C.B. (2017). Microbial vertical transmission during human pregnancy. Cell Host Microbe.

[48] Delorme-Axford E., Sadovsky Y., Coyne C.B. (2014). The placenta as a barrier to viral infections. Annu. Rev. Virol..

[49] Kreis N.N., Ritter A., Louwen F., Yuan J. (2020). A message from the human placenta: Structural and immunomodulatory defense against SARS-CoV-2. Cells.

[50] León-Juárez M., Martínez-Castillo M., González-García L.D., Helguera-Repetto A.C., Zaga-Clavellina V., García-Cordero J., Flores-Pliego A., Herrera-Salazar A., Vázquez-Martínez E.R., Reyes-Muñoz E. (2017). Cellular and molecular mechanisms of viral infection in the human placenta. Pathog. Dis..

[51] Raschetti R., Vivanti A.J., Vauloup-Fellous C., Loi B., Benachi A., De Luca D. (2000). Synthesis and systematic review of reported neonatal SARS-CoV-2 infections. Nat. Commun..

[52] Garazzino S., Montagnani C., Donà D., Meini A., Felici E., Vergine G., Bernardi S., Giacchero R., Lo Vecchio A., Marchisio P. (2020). Italian SITIP-SIP SARS-CoV-2 paediatric infection study group. Multicentre Italian study of SARS-CoV-2 infection in children and adolescents, preliminary data as at 10 April 2020. Eurosurveillance.

[53] Knight M., Bunch K., Vousden N., Morris E., Simpson N., Gale C., O’Brien P., Quigley M., Brocklehurst P., Kurinczuk J.J. (2020). Characteristics and outcomes of pregnant women admitted to hospital with confirmed SARS-CoV-2 infection in UK: National population based cohort study. BMJ.

[54] Verma S., Bradshaw C., Auyeung N., Lumba R., Farkas J.S., Sweeney N.B., Wachtel E.V., Bailey S.M., Noor A., Kunjumon B. (2020). Outcomes of maternal-newborn dyads after maternal SARS-CoV-2. Pediatrics.

[55] Dumitriu D., Emeruwa U.N., Hanft E., Liao G.V., Ludwig E., Walzer L., Arditi B., Saslaw S., Andrikopoulou M., Scripps T. (2020). Outcomes of neonates born to mothers with severe acute respiratory syndrome coronavirus 2 infection at a large medical center in New York City. JAMA Pediatr..

[56] Khoury R., Bernstein P.S., Debolt C., Stone J., Sutton D.M., Simpson L.L., Limaye M.A., Roman A.S., Fazzari M., Penfield C.A. (2020). Characteristics and outcomes of 241 births to women with severe acute respiratory syndrome coronavirus 2 (SARS-CoV-2) infection at five New York City medical centers. Obstet. Gynecol..

[57] Walker K.F., O’Donoghue K., Grace N., Dorling J., Comeau J.L., Li W., Thornton J.G. (2020). Maternal transmission of SARS-COV-2 to the neonate, and possible routes for such transmission: A systematic review and critical analysis. BJOG.

[58] Ashraf M.A., Keshavarz P., Hosseinpour P., Erfani A., Roshanshad A., Pourdast A., Nowrouzi-Sohrabi P., Chaichian S., Poordast T. (2020). Coronavirus disease 2019 (COVID-19): A systematic review of pregnancy and the possibility of vertical transmission. J. Reprod. Infertil..

[59] Schwartz D.A., Anoko J.N., Abramowitz S. (2019). Pregnant in the Time of Ebola: Women and Their Children in the 2013–2015 West. African Epidemic.

[60] Schwartz D.A. (2017). Viral infection, proliferation, and hyperplasia of Hofbauer cells and absence of inflammation characterize the placental pathology of fetuses with congenital Zika virus infection. Arch. Gynecol. Obstet..

[61] Ritter J.M., Martines R.B., Zaki S.R. (2017). Zika virus: Pathology from the pandemic. Arch. Pathol. Lab. Med..

[62] Rosenberg A.Z., Yu W., Hill D.A., Reyes C.A., Schwartz D.A. (2017). Placental pathology of Zika virus: Viral infection of the placenta induces villous stromal macrophage (Hofbauer Cell) proliferation and hyperplasia. Arch. Pathol. Lab. Med..

[63] Labarrere C., Mullen E. (1987). Fibrinoid and trophoblastic necrosis with massive chronic intervillositis: An extreme variant of villitis of unknown etiology. Am. J. Reprod. Immunol. Microbiol..

[64] Labarrere C.A., Bammerlin E., Hardin J.W., Dicarlo H.L. (2014). Intercellular adhesion molecule-1 expression in massive chronic intervillositis: Implications for the invasion of maternal cells into fetal tissues. Placenta.

[65] Bos M., Nikkels P., Cohen D., Schoones J.W., Bloemenkamp K., Bruijn J.A., Baelde H.J., van der Hoorn M., Turner R.J. (2018). Towards standardized criteria for diagnosing chronic intervillositis of unknown etiology: A systematic review. Placenta.

[66] Marchaudon V., Devisme L., Petit S., Ansart-Franquet H., Vaast P., Subtil D. (2011). Chronic histiocytic intervillositis of unknown etiology: Clinical features in a consecutive series of 69 cases. Placenta.

[67] Weber M.A., Nikkels P.G., Hamoen K., Duvekot J.J., de Krijger R.R. (2006). Co-occurrence of massive perivillous fibrin deposition and chronic intervillositis: Case report. Pediatr. Dev. Pathol..

[68] Abdulghani S., Moretti F., Gruslin A., Grynspan D. (2017). Recurrent massive perivillous fibrin deposition and chronic intervillositis treated with heparin and intravenous immunoglobulin: A case report. J. Obstet. Gynaecol. Can..

[69] Ismail M.R., Ordi J., Menendez C., Ventura P.J., Aponte J.J., Kahigwa E., Hirt R., Cardesa A., Alonso P.L. (2000). Placental pathology in malaria: A histological, immunohistochemical, and quantitative study. Hum. Pathol..

[70] Di Gennaro F., Marotta C., Locantore P., Pizzol D., Putoto G. (2020). Malaria and COVID-19: Common and different findings. Trop. Med. Infect. Dis..

